# High Proliferative Placenta-Derived Multipotent Cells Express Cytokeratin 7 at Low Level

**DOI:** 10.1155/2019/2098749

**Published:** 2019-07-15

**Authors:** V. Shablii, M. Kuchma, H. Svitina, I. Skrypkina, P. Areshkov, V. Kyryk, T. Bukreieva, V. Nikulina, Iu. Shablii, G. Lobyntseva

**Affiliations:** ^1^Institute of Molecular Biology and Genetics of National Academy of Sciences of Ukraine, Kyiv, Ukraine; ^2^Institute of Cell Therapy, Kyiv, Ukraine; ^3^State Institute of Genetic and Regenerative Medicine of Academy of Medicine of Ukraine, Kyiv, Ukraine; ^4^Coordination Centre for Transplantation of Organs, Tissues and Cells, Ministry of Health of Ukraine, Ukraine

## Abstract

The purpose of this study was to investigate the immunophenotypes and gene expression profile of high proliferative placenta-derived multipotent cells (PDMCs) population at different stages of culture. We demonstrated that the colonies resulting from single cells were either positive or negative for CK7, whereas only PDMC clones with weak CK7 expression (CK7^low^-clones) were highly proliferative. Interestingly, vimentin positive (Vim^+^) placental stromal mesenchymal cells did not express CK7* in situ*, but double CK7^+^Vim^+^ cells detection in tissue explants and explants outgrowth indicated CK7 inducible expression* in vitro*. PCNA presence in CK7^+^Vim^+^ cells during placental explants culturing confirmed belonging of these cells to proliferative subpopulation. Transcription factors CDX2 and EOMES were expressed in both CK7^low^-clones and subset of stromal mesenchymal cells of first-trimester placental tissue* in situ*. Meanwhile, CK7^low^ -clones and stromal mesenchymal cells of full-term placental tissue* in situ* expressed ERG heterogeneously.* SPP1*,* COL2A1*, and* PPARG2* mesodermal-related genes expression by CK7^low^-clones additionally confirms their mesenchymal origin. Inherent stem cell-related gene expression (*IFTM3, POU5F1,* and* VASA*) in CK7^low^-clones might indicate their enrichment for progenitors. Finally, in CK7^low^-clones we observed expression of such trophoblast-associated genes as* CGB* types I and II, fusogenic* ERVW-1*,* GCM1*, and* GATA3*. Thus, our results indicate that PDMCs acquired the representative immunophenotype signature under culture conditions.

## 1. Introduction

Human placenta contains populations of different stem cell types, namely, the mesenchymal stem/stromal cells (MSC) [1], trophoblast stem cells [2], hematopoietic stem cells [3], and endothelial progenitor cells [4]. Compared to adult MSCs, placental ones possess advantages for regenerative medicine as faster kinetics, greater expansion potential, wider differentiation potential [5], and lower immunogenicity [6].

Based on pericyte markers CD146 and NG2 expression, the perivascular origin of foetal placental MSCs has been hypothesized [1, 3]. Additionally, the vascular origin of MSCs isolated from* decidua basalis *was confirmed by Kusuma et al., reporting presence of MSC markers 3G5, alpha-smooth muscle actin (*α*SMA), STRO-1, and FZD-9 around* decidua basalis* vessels ranging in size from 50 mm to 100 mm [7].

The different mesenchymal zones of the umbilical cord, such as Wharton's jelly zone, subamnioblastic zone, and subvascular zone, were characterized by expression of cytokeratins (CK) 7, 18, and 19 [8]. Along with aforementioned CK7 may be a marker of early mesoderm cells in extraembryonic tissues since the CK7^+^ mesodermal cells appeared after treatment of human ESCs with BMP4 and Activin/Nodal receptor inhibitor SB431542 [9]. Interestingly, CK7 was expressed in both adult hematopoietic stem cells and fetal liver (CD150^+^KSL) ones* in vivo *[10]. Furthermore, other mesodermal progenitors as fetal liver hepatic stellate cells [11] and mesothelial cells express the CK7 [12]. Therefore, mesenchymal stromal cells differ in expression of cytokeratins. Based on the heterogeneity of the placenta-derived multipotent cells (PDMCs) the expression of cytokeratins in the subset of placental mesenchymal progenitors remains unclear.

In addition to CK7, PDMC express other trophoblast-associated genes, namely, glial cell missing-1 (*GCM1*) and human chorionic gonadotropin alpha (*αCG*) [9], but, in contrast to trophoblasts, they are positive for vimentin (VIM) [13] and *α*SMA [1]. In spite of this, a large number of authors consider isolated cells* in vitro* as trophoblasts based on the detection of a limited list of trophoblast-associated genes: СК7, CK18, human chorionic gonadotropin beta (*CGB*), fusogenic gene* ERVW-1*, and transcription factor* GCM1 *[14].

We assume novel study of trophoblast- (*GCM1*,* GATA3, ERVW1*, and* CGB*), mesenchymal- (*ERG, PPARG2, COL2A1, SPP1, *Vim, and *α*SMA), and stemness-related (*POU5F1, IFTM3, *and* VASA) *markers expression in PDMCs and PDMC-derived clones at different stages of culturing may prompt the additional markers usage for evaluation of both trophoblast and MSCs culture purity.

Proceeding from the fact that development of the placenta is regulated by caudal type homeobox 2 (*CDX2*), eomesodermin (*EOMES*), and POU class 5 homeobox 1 (*POU5F1)* transcription factors [15–18], we decided to focus on their expression in PDMCs and PDMC-derived clones.

## 2. Materials and Methods

This study and the consent procedure were approved by the Committee of Human Research of the Institute of Cell Therapy (#2-13).

Standard methods of adipogenic and osteogenic differentiation of PDMCs; RNA extraction; RT-PCR; karyotyping; and list of used primers and antibodies can be found in Supplementary Materials.

### 2.1. Isolation and Culture of PDMC

Term placentas (n=15; delivered after clinically normal pregnancies or Caesarean section) were collected from 23- to 36-year-old donors at 39–41 weeks of gestation in the Kyiv city maternity hospital #3. First-trimester placentas (n=7) were obtained from elective aborted human foetuses at 6 to 12 weeks of gestation with the women's written informed consent (City Clinical Hospital #2, Kyiv). All donors provided written informed consent for the sourcing and the usage of their placentas and aborted foetal tissues for the approved study.

The amnion was removed, and an approximately 4 g fragment of chorionic plate and chorionic villus (3–7 mm thick) was cut off with scissors. The tissue fragment was minced into small pieces (1–3 mm) and washed intensively on a shaker in Hanks' balanced salt solution (HBSS) (HyClone, USA) supplemented with penicillin (100 U/ml) and streptomycin (50 mg/ml) until the washing solution became colourless. Then, the fragments were digested with 0.1% collagenase I (Serva, Germany) and 0.6 U/ml dispase I (Gibco, USA) in 5 ml of DMEM (HyClone, USA) with 5 mM HEPES (MP Biomedicals, USA). Semidigested pieces of tissue were seeded into alpha-MEM (HyClone, USA) with an addition of 15% FBS (HyClone, USA), 1 × RPMI amino acid solution (Sigma, USA), and 1× streptomycin/penicillin (Sigma, USA), which completed the cultural medium. These explants were cultivated in cell culture flasks on an adhesive surface (Sarstedt, Germany) at +37°С and 5% СО_2_. Culture medium was changed twice a week. For immunohistochemistry the attached full-term placental tissue explants (FTPE) at 10 days were fixed in 4% PFA for 15 min at RT. When outgrowth of cells reached 80–90% confluence in a monolayer, they were detached using 0.05% trypsin and 0.02% EDTA (Biochrom, UK), washed, counted, and passaged at the inoculation density of 4–5 ×10^3^ cells/cm^2^ on culture-treated surface plastic flasks, referred to as passage 1 (P1). PDMCs between the first and seventh passages were used for analysis (P1 through P7).

### 2.2. Obtaining PDMC Clones by Subsingle Cell Seeding

Every PDMCs suspension (from 4 individual donors) was diluted serially in alpha-MEM, and the last dilution corresponding to the concentration of 1 cell per 200 *μ*l in complete medium was placed into each well of a 96-well tissue culture plate (Sarstedt, Germany) at the volume 100 *μ*l. The culture medium was replaced at the 14^th^ day of culturing. At the 3^rd^, 7^th^, 14^th^, and 21^st^ days in culture, each well was observed by phase-contrast light microscopy to control the quality of cells. Only wells with single colonies of cells observed at day 3 and day 7 were maintained further by replacement of culture medium. Some colonies were isolated and subcultured. The majority of clones at P0 were seeded in two wells; one was used for immunocytochemistry detection of CK7, while another for further cell expansion. Several clones with high proliferative potential were propagated until cell senescence was observed or cells stopped proliferating. Immunocytochemistry was performed at all passages; gene expression and differentiation potential were tested at P5.

### 2.3. Flow Cytometry

Both PDMCs (from 10 individual donors) and single cell-derived clones (from 5 individual donors) were washed with cold Cell Wash buffer (Becton Dickinson, USA) and incubated for 30 min at +4°C with fluorochrome-conjugated monoclonal antibodies (Table S2) at an appropriate dilution of 0.5 *μ*g per 10^6^ cells.

Unbound primary antibodies were washed away with Cell Wash buffer. The samples were acquired on a BD FACSAria cell sorter (Becton Dickinson, USA) and analyzed using BD FACSDiva 6.1.2 software. To adjust the compensation settings of fluorochromes overlapping control samples were used: unstained control, single stained, and fluorescence minus one control.

### 2.4. Quantitative Real-Time RT-PCR

The relative gene expression level was conducted using a iCycler СFX96 Real-Time PCR system (Bio-Rad, USA) at standard conditions. Each qPCR reaction contained 1.5 *μ*l cDNA, 0.3 *μ*M of each specific primer (Table S1), and 1 × Maxima SYBR Green qPCR Master Mix (Thermo Scientific, USA) according to the manufacturer's recommendations. PCR was performed under the following conditions: 10 min at 95°C, followed by 40 cycles of 15 s at 95°C, and 1 min at 60°C. No-template controls (NTCs) were used as negative controls. The specificity of the PCR primers was verified by melting curve and agarose gel analyses. If the difference between duplicate samples was greater than one cycle threshold the analysis was repeated. Obtained results from at least three separate experiments were normalized to the reference gene *β*-actin (*ACTB*). The relative levels of genes expression values were calculated using the 2^–ΔΔCt^ method, where ΔΔCt=ΔCt_target_–ΔCt_ACTB_ [19].

### 2.5. Immunocytochemistry

PDMCs from individual donors (n=4) were grown on glass slides (Nunclon™Δ Surface, Sigma, USA), fixed in 4% PFA for 15 min at RT, and permeabilized in acetone/methanol solution (v/v 1:1) at -20°С for 40 min. Endogenous peroxidase activity was inhibited by incubation with 0.3%  Н_2_О_2_ for 5 min. Nonspecific antibodies binding was blocked by 0.5% BSA in 0.1 М PBS. Primary antibodies (Table S3) were applied in Antibody Diluent (S0809, Dako, Denmark) at +4°C overnight.

Visualization of specific binding was performed with the Mouse/Rabbit PolyVue HRP/DAB Detection System (Diagnostic BioSystems, USA). Images were captured using an inverted microscope (Olympus IX71, Olympus Corporation, Japan) at 50× magnification.

### 2.6. Immunofluorescence

PDMCs from individual donors (n=10) were fixed and permeabilized as described above. Nonspecific binding of antibodies was blocked by 0.5% BSA in 0.1 М PBS. After incubation with specific primary antibodies in PBS at +4°C overnight, appropriate Alexa 488 or Alexa 555 conjugated secondary antibodies (Table S3) were added for 1 h at RT. Confocal analysis was performed with a Zeiss LSM 510 Meta microscope (Carl Zeiss Microscopy GmbH, Germany) and images were captured with Zeiss LSM Image Browser software.

### 2.7. Immunohistochemistry Fluorescence

First-trimester placental tissue (FiTPT) at 7–11 weeks of gestation (n=7) and full-term placental tissue (FTPT, n=15) were fixed in 4% PFA for 24 h, embedded in paraffin, and sectioned according to standard histology methods. Paraffin-embedded tissues were deparaffinized in 2 changes of xylene and rehydrated in decreasing concentrations of ethanol.

Antigen retrieval was performed in Tris-EDTA buffer (10 mM Tris Base, 1 mM EDTA solution, pH 9.0) at +95°С for 30 min. Nonspecific reactivity was reduced by incubating tissue sections in blocking solution (0.5% BSA and 1% goat serum in 0.1 M PBS with 0.3% Triton X-100) for 30 min.

Immunostaining was accomplished by overnight incubation at +4°С with primary antibodies (Table S3) diluted in Antibody Diluent (Dako, Denmark). After extensive washing with 0.1 M PBS, samples were incubated in the appropriate species-specific secondary antibodies (Table S3) diluted 1:1000 in Antibody Diluent (Dako, Denmark) for 1 h at RT in the dark. After nuclear staining with Hoechst 33258 (Vector Laboratories, UK), slides were mounted in Mowiol® 4-88 (Sigma, USA).

Confocal analysis was performed with a Zeiss LSM 510 Meta microscope (Zeiss, Germany) and images were captured with Zeiss LSM Image Browser software.

### 2.8. Fluorescence* In Situ* Hybridization (FISH)

PDMCs from individual donors (n=4) were fixed on slides and digested with proteinase K before hybridization with human specific centromeric probe CEPХ SpectrumGreen probe and CEPY SpectrumOrange probe (Abbot Molecular, USA) according to the manufacturer's protocol. Nuclei were counterstained with DAPI and viewed under an Olympus IX 71 fluorescence microscope (Olympus Corporation, Japan). A total of 500 cells per slide were analyzed.

### 2.9. Statistical Analysis

Results are represented as the means ± standard error for normally distributed data or medians with ranges for nonnormally distributed data. The significant differences between groups were assessed by two-tailed Student's t-test or Mann–Whitney U-test, whenever applicable. P-values of <0.05 were considered to be significant.

## 3. Results

### 3.1. PDMCs Were of Foetal Origin

**Figure 1:**
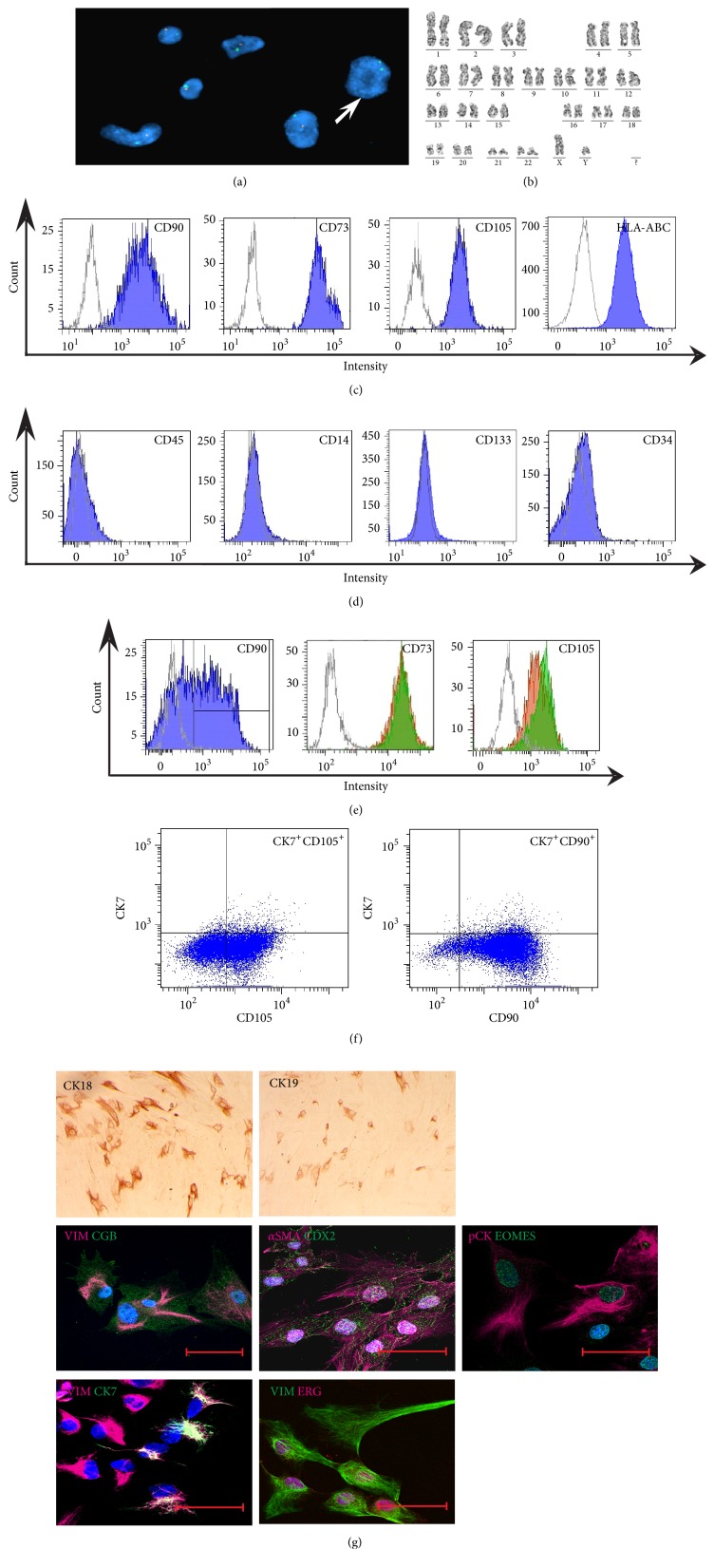
Characteristics of PDMCs. (a) The representative example of FISH analysis demonstrated the presence of a few maternal cells in PDMCs; for X (green) and Y (orange) chromosomes, the arrow indicates the maternal cell nucleus; ×1000. (b) A representative example of normal male karyotype of a PDMC line at P3 (n=4). (c) PDMCs were positive for CD90, CD73, CD105, and HLA-ABC at P3. (d) PDMCs were negative for CD34, CD14, CD45, and CD133 at P3. (e) Bimodal character of CD90 expression and expression of CD73 and CD105 on the CD90^+^ (red) and the CD90^−^ (green) subpopulations of the PDMCs at P3. Cells stained by the corresponding antibodies are indicated by filled histograms; isotype controls are indicated by open grey histograms. (f) CK7-positive PDMCs expressed CD90 and CD105 at P3. (g) PDMCs were positive for the mesenchymal markers VIM and *α*SMA, and epithelial-associated proteins pCK, CK7, CGB, CK18, and CK19 at P3; brown colour – positive cells; ×50. The transcription factors CDX2, EOMES, and ERG were detected in PDMCs at P3. Scale bar: 50 *μ*m. Because of high complexity and for better representation of multicolour confocal imaging, we only show merged images. Individual pictures of each colour channel can be obtained on request.

For more relevant interpretation of results, foetal cells of both sexes (n=10, 4 males and 6 females) were used in the research. FISH analysis confirmed the foetal origin of investigated cells, showing that all PDMCs from male newborns contained X and Y chromosomes in nuclei; the level of contamination by maternal cells was approximately 0.85% (0–3%, n=4) at P3 (Figure 1(a)). Chromosomal analysis of PDMCs populations (n=10) did not show any chromosomal abnormalities. All tested cells had normal karyotype, either male (46, XY) or female (46, XX) (Figure 1(b)).

### 3.2. PDMCs Expressed Proteins Typical of Trophoblast and Mesenchymal Cells

The flow cytometry analysis demonstrated that PDMCs (n=10) from P1 to P6 were positive for CD90, CD73, CD105, and HLA-ABC (Figure 1(c)) and negative for CD34, CD45, CD133, and CD14 (Figure 1(d)). However, the level of CD90 expression fluctuated from 73.8% to 98.3%. Some of the PDMC cultures (n=3) possessed bimodal patterns of CD90 expression and comprised both CD90^+^ and CD90^−^ populations (Figure 1(e)). Multiparameter flow cytometry demonstrated a similar level of CD73 and CD105 expression on the cell surface of both CD90^−^ and CD90^+^ subpopulations (Figure 1(e)).

PDMCs contain CK7^+^ cell population. Despite the fact that CK7-positive cytotrophoblasts are characterized by the lack of CD90 expression, almost all of CK7^+^ PDMCs (n=4) were positive for CD90 (Figure 1(f)). Additionally, multicolour flow cytometry of PDMCs showed expression of CD90 and CD105 on CK7^+^ cells (Figure 1(f)).

All PDMCs (n=10) simultaneously expressed VIM and pan-cytokeratin (pCK) during six passages of cultivation (Figure 1(g)). PDMCs (n=4) were heterogeneous for the expression of the epithelial cytoskeleton proteins CK18 (38%) and CK19 (31%) (Figure 1(g)), as well as the mesenchymal marker *α*SMA (65%) (Figure 1(g)).

The percentage of double positive cells for VIM and CK7 (Figure 1(g)) decreased significantly (p<0.05) from 37.6% (26.6–49.4%, n=6) at P1 to 13.4% (2.5–31.1%, n=6) at P3. We found that all PDMCs within six passages were positive for another trophoblast marker chorionic gonadotropin beta (CGB), which colocalized with VIM (Figure 1(g)).

All PDMCs (n=6) expressed CDX2 and EOMES, transcription factors equally critical for trophoblast and mesoderm development (Figure 1(g)). Both factors were localized predominantly in the cell nuclei, but a weak staining signal was also observed in the cytoplasm of some cells. Furthermore, we detected the nuclear localization of Ets-related gene product (ERG, Figure 1(g)) in more than 80% of PDMCs at P3 (n=5).

### 3.3. PDMCs Differentiated into Mesoderm Lineages

**Figure 2:**
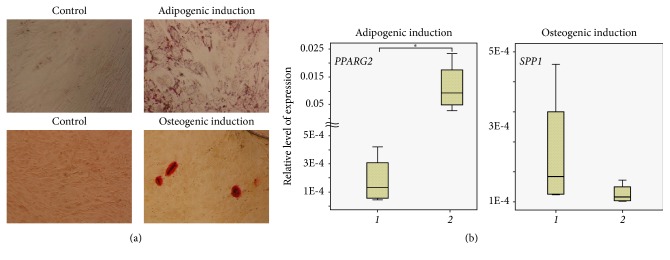
Multipotent potential of PDMCs. (a) Abundance of lipid vacuoles and single nodules of extracellular calcium matrix were detected in PDMC cultures at P3 after adipogenic and osteogenic induction; Oil Red O and Alizarin Red S staining, ×50. (b) The expression level of* PPARG2* significantly increased in contrast to the slightly decreasing level of* SPP1* expression in PDMCs at P3 under adipogenic and osteogenic induction conditions, respectively; n=4, ∗: p<0.05, 1 – control, 2 – differentiation.

We have shown that PDMCs (n=4) at P3 had low capacity for osteogenic differentiation compared to adipogenic differentiation under appropriate induction conditions. Scattered nodules of extracellular calcium matrix were detected in PDMC cultures after osteogenic induction. In addition, the level of osteopontin expression under induction conditions did not differ in comparison to control (Figures 2(a) and 2(b)).

Analysis revealed the production of Oil Red O-positive lipid vacuoles in PDMCs when they were cultivated in adipogenic permissive medium for three weeks, and real-time PCR analysis showed significantly increased expression of adipocyte differentiation regulator* PPARG2* (Figures 2(a) and 2(b)).

### 3.4. Characteristics of the PDMCs-Derived Clones

**Figure 3:**
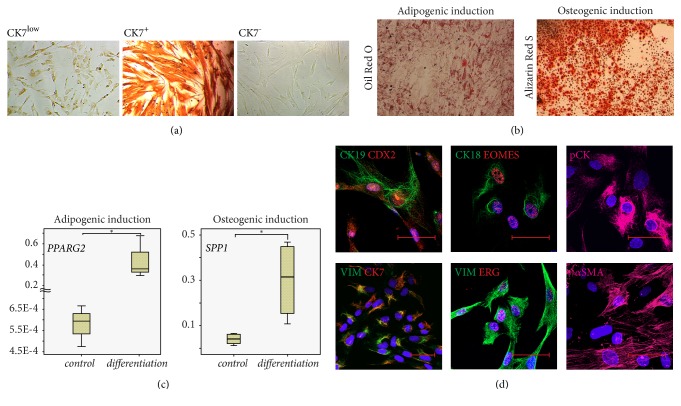
Characteristics of the single-cell-derived clones. (а) Three types of PDMCs-derived clones; immunodetection of CK7, brown colour – positive cells; ×100. (b) CK7^low^-clones accumulated lipid inclusions and calcium-containing extracellular matrix under adipogenic and osteogenic conditions, respectively; Oil Red O and Alizarin Red S staining, ×50. (c) The level of* PPARG2 *and* SSP1* expression increased in CK7^low^-derived clones at P5 under adipogenic and osteogenic induction conditions, respectively; n=4, ∗: p<0.05. (d) Representative immunocytochemistry of CK7^low^-derived clone PDMC-C1 at P4.

A total of 19 clones were isolated from three placenta samples and were either positive or negative for CK7 (Figure 3(a)). The percent of single cell-derived clones with low expression of CK7 (CK7^low^-clones), CK7^+^ clones and CK7^−^ clones is 52% (n=10), 27% (n=5), and 21% (n=4), respectively.

CK7^low^-clones had the similar or higher proliferative characteristics in comparison to bulk PDMCs during more than ten passages (Figure S2A). It should be noted that at the 10^th^ passage the estimated total cell harvest ranged from 51.45×10^6^ to 118.4×10^9^ from CK7^low^-clones (Figure S2B). In contrast, both CK7^+^ and CK7^−^ clones stopped to proliferate at P1. All clones lost the expression of CK7 at different times of propagation after the 3^rd^ passage.

All CK7^low^-clones at P5 had the capacity to differentiate into adipogenic and osteogenic lineages (Figure 3(b)). The expression levels of adipogenesis- or osteogenesis-related genes,* PPARG2* and* SPP1*, correspondingly, were significantly increased under appropriate differentiation conditions (Figure 3(c)).

The CK7^low^-clones (n=10) possessed CD90^+^CD73^+^CD105^+^CD44^+^CD45^−^CD34^−^ surface immunophenotype (Figure S3) and expressed the mesoderm-related cytoskeleton proteins VIM and *α*SMA (Figure 3(d)). pCK was expressed in all CK7^low^-clones, but the expression of CK18 and CK19 was heterogeneous (Figure 3(d)). The transcription factors CDX2 and EOMES were detected in all CK7^low^-clones. In turn, we detected CDX2 in the nuclei of all cells during P3-P10 (Figure 3(d)). In addition, the expression of EOMES was positive in CK7^low^-clones with immunofluorescence signal in the nuclei of all cells during P3-P10 (Figure 3(d)). The Ck7^low^-clones were either positive (n=7) or negative (n=3) for ERG with strong nuclear localization (Figure 3(d)).

We detected expression of extraembryonic mesoderm-associated transcription factors* CDX2*,* EOMES* in all clones, but stem cells-related genes* POU5F1 *and* VASA *in CK7^low^-clones were expressed heterogeneously (Table S4); only three clones (PDMC-C1, PDMC-C6, and PDMC-C7) expressed all of these genes (Figure S1A). In contrast to* VASA*, another primordial germ cell marker,* IFITM3*, was common for all clones. Analysis of trophoblast-related genes (*GCM1*,* GATA3*,* ERVW1*, and* CGB*) expression profile (Figure S1A, Table S4) revealed* GCM1*,* ERVW1*, and* GATA3 *mRNAs were detected only in some CK7^low^-clones. The sequencing analysis showed that* CGB *in CK7^low^-clones was represented by either nontrophoblastic (type I) transcript type or type II (trophoblast-specific) transcripts (Figure S1B). Mesodermal-related genes* SPP1*,* COL2A1*, and* PPARG2* were expressed in all CK7^low^-clones (Table S4).

### 3.5. FiTPT and FTPT Contained Cells with the Immunophenotype Similar to PDMCs

**Figure 4:**
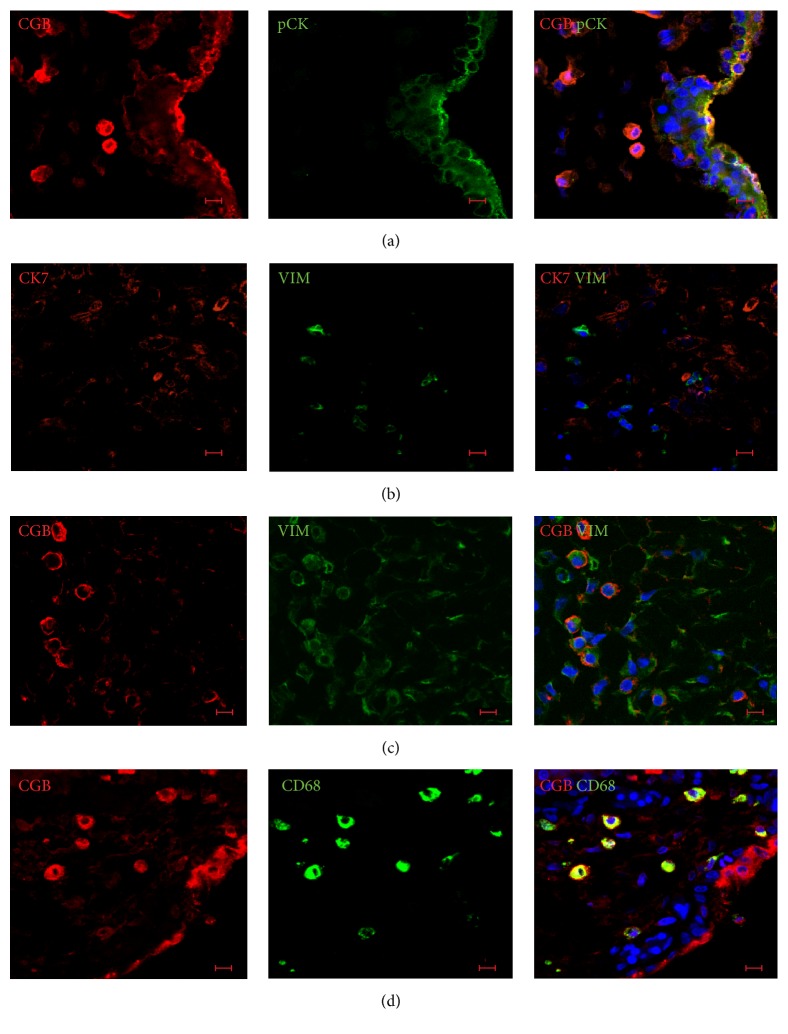
Localization of PDMC markers in the placental chorionic villi. (a) Trophoblast cells coexpressed pCK and CGB. (b) VIM and CK7 were expressed in different placental cell populations. (c) Some stromal placental cells had PDMC-like VIM^+^CGB^+^-immunophenotypes. (d) CGB^+^-stromal cells were positive for macrophage marker CD68. Representative immunofluorescence of FiTPT (a, c, d) and FTPT (b); scale bar: 10 *μ*m.

To investigate the origin of PDMCs, we performed immunohistochemistry on FiTPT, FTPT, and FTPE. Most of the cytokeratin-positive cells expressed CGB in FiTPT, in contrast to FTPT, where low levels of immunostaining signals for CGB were observed (Figure 4(a)).

Common mesenchymal marker VIM was detected in the stromal cells of chorion villi of FTPT and FiTPT, and all of them did not express CK7 (Figure 4(b)).

**Figure 5:**
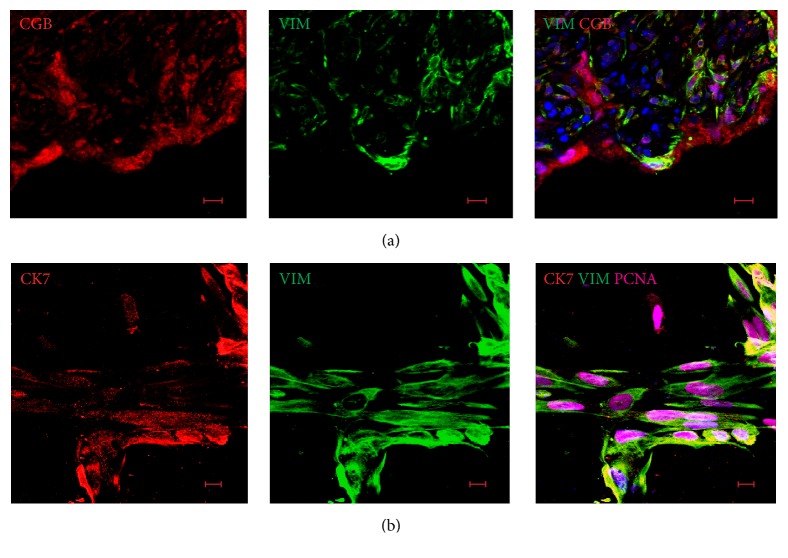
Immunophenotype of stromal cells in FTPE. (a) Placental stromal cells expressed CGB. (b) CK7^+^VIM^+^ cells located on the periphery of explants. Scale bar: 10 *μ*m.

Although most CGB-positive cells did not express VIM, we detected some CGB^+^VIM^+^ cells in the stromal part of villous chorion of FTPT and FiTPT (Figure 4(c)). The subsequent experiments showed that all stromal CGB-positive cells were immunoreactive for CD68 antibodies, suggesting that VIM^+^CGB^+^ stromal cells were represented by a tissue-specific macrophage population distinct from PDMCs (Figure 4(d)). Therefore, stromal nonhematopoietic cells with expression profiles similar to PDMCs were not detected in FTPT and FiTPT* in situ*. We demonstrated that cells with PDMC-like immunophenotypes arose in FTPE. Indeed, all VIM^+^ cells in FTPE coexpressed CGB (Figure 5(a)). Additionally, VIM^+^CK7^+^ were detected in cells outgrowth from explants and were positive for proliferation marker PCNA (Figure 5(b)).

**Figure 6:**
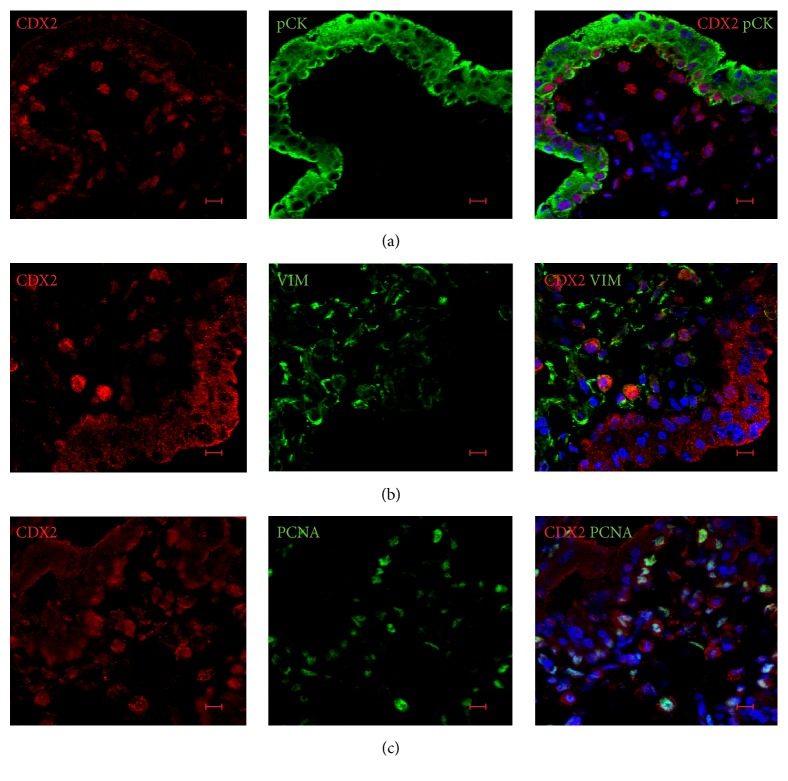
The characteristics of CDX2 expression in FiTPT. (a) CDX2 was expressed in cytotrophoblasts of FiTPT. (b) Some stromal cells of FiTPT expressed CDX2. (c) The majority of CDX2^+^ cells coexpressed PCNA in FiTPT. Scale bar: 10 *μ*m.

CDX2 was not detected in FTPT* in situ*. In the FiTPT, this transcription factor was strongly expressed in the nuclei of some cytotrophoblast cells (Figure 6(a)), and comparatively weakly in either cytoplasm or both cytoplasm and nucleus of stromal cells and syncytiotrophoblasts, depending on tissue sample (Figures 6(a) and 6(b)).

**Figure 7:**
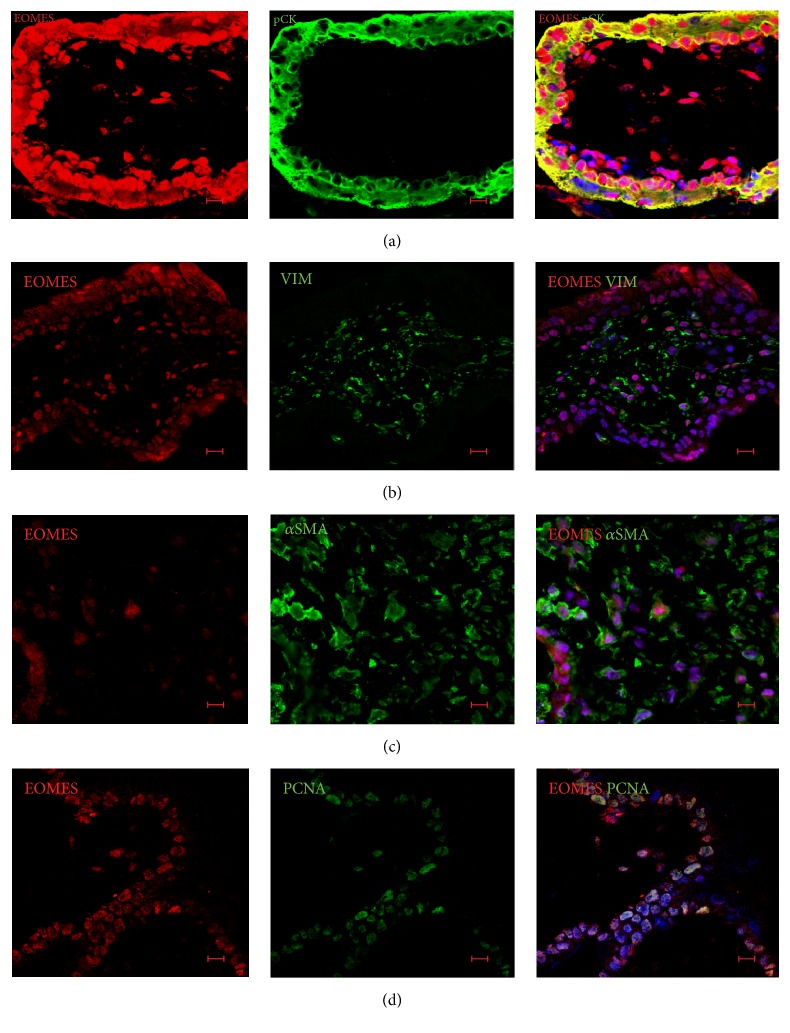
The character of EOMES expression in FiTPT. (a) EOMES was expressed in stromal cells and both cytotrophoblasts and syncytiotrophoblasts of FiTPT. (b) EOMES^+^ villous stromal cells were VIM-positive. (c) Some *α*SMA^+^ cells coexpressed EOMES. (d) The majority of PCNA^+^ cells coexpressed EOMES. Scale bar: 10 *μ*m.

Notably, cell-type-specific expression of CDX2 was confirmed by detection of trophoblast (Figure 6(a)) and mesenchymal (Figure 6(b)) cell markers, pCK and VIM, correspondently. In double labelling for CDX2 and PCNA, we observed that CDX2^+^ cells were either positive or negative for this proliferation marker (Figure 6(c)). EOMES was observed in stromal cells and both cytotrophoblasts and syncytiotrophoblasts of FiTPT (Figure 7(a)).

Indeed, we found that EOMES-positive villous stromal cells were also positive for either VIM (Figure 7(b)) or *α*SMA (Figure 7(c)). EOMES was localized either in the cytoplasm or in both cytoplasm and nucleus of trophoblasts and stromal cells. Using dual labelling for EOMES and PCNA, we found that PCNA was confined to a subset of EOMES-positive cells (Figure 7(d)).

**Figure 8:**
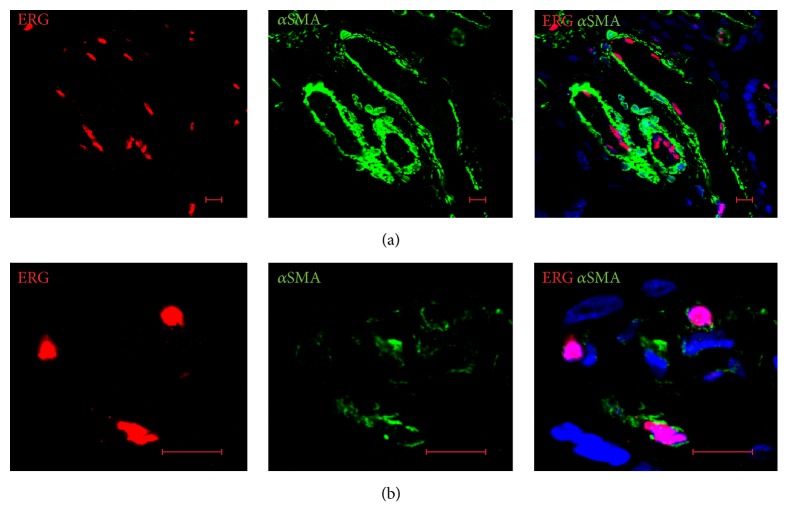
ERG is expressed in the stromal part of placental tissue. (a) ERG-positive cells were found within the endothelial cells of placental tissue. (b) Single villous stromal cells were double positive for ERG and *α*SMA. Representative immunofluorescence of FTPT. Scale bar: 10 *μ*m.

ERG-positive cells were found within the endothelial cells of FTPT and FiTPT (Figure 8(a)).

Using dual labelling for ERG and *α*SMA, we found that ERG was mostly absent in a subset of *α*SMA-positive cells, but in rare examples villous stromal cells were coexpressed both in FiTPT as well as in FTPT (Figure 8(b)).

Thus, we showed that CDX2 and EOMES were expressed in mesodermal and trophoblast cells of FiTPT, including proliferated stromal cells; ERG production in some stromal mesodermal nonendothelial cells in both FiTPT and FTPT; and inducible expression of several epithelial markers acquired under culture conditions* ex vivo*.

## 4. Discussion

Some PDMC lines showed bimodal distribution of CD90 expression. This phenomenon was previously observed [20, 21]. The expression of epithelial markers such as cytokeratins (pCK, CK18, CK19, and CK7) is likely to be a unique feature of PDMCs acquired under culture conditions. Although we showed that cytokeratins appeared in PDMCs* in vitro*, their expression was previously detected in umbilical cord-derived MSCs and in stromal cells of different compartments of umbilical cord* in situ* [22].

This study showed that both CK7^+^ and CK7^−^ subpopulations of self-renewing PDMCs had low proliferative potential compared to cells with low expression of CK7. Indeed, clones that were negative for CK7 or strongly expressed this protein were not maintained in cell culture during passaging. Our presented data demonstrated the CK7 as monomers or granules in CK7^low^-clones in contrast to intermediate filament in CK7^+^ clones. Similar monomeric structure of cytokeratins is inherent in mitotic cells as shown by Toivola* et al.* [23]. However, it is interesting that the high proproliferative clones terminated the expression of CK7 after the third passage, which proves the unimportance of CK7 to maintain proliferative properties. Also, hematopoietic cells lost the expression of CK7 at early stages of differentiation, herewith they maintain the proliferation capacity [10]. Mesodermal origin mesothelium has two cell types, epithelial (mesothelial layer, expressing CK7) and fibroblast-like (subepithelial layer, lacking CK7). During mesothelial-mesenchymal transition epithelial markers are lost, including CK7, and cells acquiring the proliferative capacity [12, 24]. Choi et al. [25] showed that freshly isolated rat quiescent hepatic stellate cells expressed some epithelial markers, including E-cadherin, СК7, and СК19, which were downregulated during culture-induced transition to myofibroblastic hepatic stellate cells. Therefore, CK7 is inherent in various mesodermal progenitors but unimportant to maintain their proliferative capacity. We assume that CK7 expression at low level is characterized for placental mesenchymal progenitors at transition from quiescent to mitotic status rather than for maintenance of proliferation.

The belonging of CK7^+^Vim^+^ cells to a proliferative PDMCs subpopulation was confirmed by expression of PCNA in these cells during placental explants culture. The stromal mesenchymal (Vim^+^) cells did not express CK7* in situ*, but double positive CK7^+^Vim^+^ cells were detected in tissue explants and explants outgrowth; hence, we conclude that expression of CK7 is induced* in vitro*. Moreover, the expression of CK7 was observed in the primary culture of amnion-derived MSCs and umbilical stromal cells* in situ* and* ex vivo *[22, 26]. To date, there is no data about the role of CK7 in the stem and progenitor cells of the mesodermal origin, nor the mechanism known to regulate the expression of CK7. It is known that the CK7 promoter contains PPAR response elements; at the same time in work by Ulrich et al. [27] and in our study the expression of this transcriptional factor was shown in PDMC. Induction PPAR*γ* expression in mesenchymal placenta-derived cells occurred under culture conditions since in stromal cells PPAR*γ* is not detected* in situ* [28, 29]. Thus PPAR*γ* could be prominent trigger for CK7 expression during PDMC culture establishing.

In the present study, the expression of* CDX2* and* EOMES* in the CK7^low^-clones was shown. In addition, we showed the expression of CDX2 and EOMES in proliferating subsets of chorionic mesenchymal (Vim^+^) cells of FiTPT* in situ*. The role of EOMES and CDX2 in the extraembryonic mesoderm development in humans is not studied sufficiently, but it is known that, in mice, CDX2 plays an essential role in posterior (somatic and extraembryonic) mesoderm development [30]. Thus, we conclude that CDX2 and EOMES are associated not only with trophoblast progenitors but also with mesenchymal progenitors of FiTPT* in situ*.

We observed either ERG-positive or -negative clones in the PDMC. Additionally, ERG was detected in both blood vessels and extravascular stromal cells of human FTPT and FiTPT. Although ERG mutation results in abnormal placenta development, including defective vasculature in the labyrinth region in mice [31], the role of this transcription factor in extraembryonic mesoderm development is insufficiently studied currently. We hypothesized that ERG expression in the PDMCs may attribute PDMCs to vasculogenic stromal cell population.

CK7^low^-clones expressed mesodermal-related genes* SPP1, COL2A1*, and* PPARG2 *[32], which additionally confirms their mesenchymal origin. Interestingly, these cells had inherent expression of the stem cell-related genes* IFTM3* and* VASA*. Earlier,* IFITM3* expression was already established in chorionic stem cells, but the expression of another gene involved in the maintenance of PGCs,* VASA*, was not observed in chorionic stem cells [33].* VASA *is important for preserving totipotency by inhibiting the expression of genes that lead to somatic differentiation [34]. Also CK7^low^-clones expressed pluripotency marker* POU5F1 *that is common for pluripotent stem cells [35], and multipotent ectoderm [35], endoderm, and mesoderm precursors [14]. Detection of* POU5F1, IFTM3,* and* VASA *at the same time can testify that the CK7^low^-cell population is enriched in progenitors.

We have demonstrated that PDMCs and CK7^low^-clones expressed the following trophoblast-associated genes*: GATA3, GCM1*,* ERVW1*, and* CGB. *The role of these genes in PDMCs is unknown, but it is clear that they cannot be used as specific markers of trophoblast subpopulations in cell culture. Our data is consistent with Kumar* et al.* [36] which confirmed* GATA3* expression in mesodermal progenitors, despite the fact that* GATA3* is known to regulate trophoblast development [37]. Also, based on our results, we concluded that it is always necessary to investigate the expression of mesenchymal-related genes in trophoblast primary culture to avoid misinterpretations.

Therefore, a high-proliferative subpopulation of PDMCs weakly expresses CK7 and these cells acquire a specific immunophenotype because of their introduction into the cell culture.

## 5. Conclusions

Human PDMCs are a heterogeneous population of cells that possess immunophenotype and differentiation potential inherent in MSCs but, at the same time, express many genes commonly thought to be trophoblast associated. The subpopulation of PDMC with high proliferation potential and capacity to differentiate in mesodermal directions weakly expressed CK7. The transcription factors ERG, EOMES, and CDX2 are typically expressed in PDMCs and placental mesenchymal progenitors* in situ*. The PDMCs acquired its immunophenotype under culture conditions.

## Data Availability

The data used to support the findings of this study are included within the article.

## Abbreviations

PDMC: Placenta-derived multipotent cells
FTPT: Full-term placental tissue
FiTPT: First-trimester placental tissue
FTPE: Full-term placental tissue explants
MSC: Mesenchymal stem/stromal cells
*CDX2*: Caudal type homeobox 2
*EOMES*: Eomesodermin
*ERVW1*: Endogenous retrovirus group W member 1
*GCM1*: Glial cell missing -1
*GATA3*: GATA binding protein 3
*POU5F1*: POU class 5 homeobox 1
*SPP1*: Secreted phosphoprotein 1
*PPARG2*: Peroxisome proliferator activated receptor gamma
*COL2A1*: Collagen type II alpha 1 chain
*IFTM3*: Interferon induced transmembrane protein 3
*VASA (DDX4)*: DEAD-box helicase 4
*CGB*: Chorionic gonadotropin beta subunit
*PCNA*: Proliferating cell nuclear antigen
ERG: Ets-related gene product
CK7: Cytokeratin 7
Vim: Vimentin
*α*SMA: Alpha-smooth muscle actin.

## Supplementary Materials

Supplementary Materials Protocols of adipogenic and osteogenic differentiation, RNA extraction, RT-PCR, and karyotyping assays are provided. Table S1: Primers sequences and product sizes. Table S2: Antibodies used for cell characterization by flow cytometry. Table S3: Antibodies used for cell characterization by immunofluorescence. Figure S1: CK7^low^-clones expressed stemness-, mesoderm-, and trophoblast-related genes. Figure S2: Proliferative characteristics of CK7^low^-single cell-derived clones. Figure S3: CK7^low^- single cell-derived clones were positive for CD90, CD73, CD105, and CD44 and negative for CD34 and CD45 at P3. Table S4: The gene expression profile of CK7^low^-clones at P5.
