# Supplementary material for: High Proliferative Placenta-Derived Multipotent Cells Express Cytokeratin 7 at Low Level
Source: Biomed Res Int. 2019 Jul 15;2019:2098749. doi: 10.1155/2019/2098749 (PMC6662495; doi:10.1155/2019/2098749)
Supplement: Supplementary Materials — Protocols of adipogenic and osteogenic differentiation, RNA extraction, RT-PCR, and karyotyping assays are provided. Table S1: Primers sequences and product sizes. Table S2: Antibodies used for cell characterization by flow cytometry. Table S3: Antibodies used for cell characterization by immunofluorescence. Figure S1: CK7low-clones expressed stemness-, mesoderm-, and trophoblast-related genes. Figure S2: Proliferative characteristics of CK7low-single cell-derived clones. Figure S3: CK7low- single cell-derived clones were positive for CD90, CD73, CD105, and CD44 and negative for CD34 and CD45 at P3. Table S4: The gene expression profile of CK7low-clones at P5. [file 2098749.f1.pdf]

Supplementary material

Adipogenic differentiation

For adipogenic differentiation, cells were grown to 90% confluency and then treated with adipogenic differentiation medium containing DMEM high glucose (Sigma, USA), 10% FBS (Gibco), 2 mM L-glutamine (Biomedicals), 5 mM HEPES (Biomedicals), 1 μM dexamethasone (Sigma, USA), 0.5 mM 3-isobutyl-1-methylxanthine (Sigma, USA), 0.06 mM indomethacin (Sigma, USA), 5 μg/ml insulin (Biochrom), 100 U/ml penicillin, and 50 μg/ml streptomycin (Sigma, USA). The medium was replaced twice weekly. After 21 days of incubation in the adipogenic induction medium, this medium was aspirated and the cells were washed with PBS. Cells were fixed with 4% paraformaldehyde (PFA) and washed with sterile water. Next, the cells were incubated with 60% isopropanol at RT for 5 min and dried. The cultures were incubated with Oil Red O (Sigma, USA) solution (0.5% vol/vol in isopropanol) for 20 min at RT. After staining, the cells were washed with distilled water before microscopy. In addition, PDMCs were pelleted, and total RNA was collected after 21 days for adipogenic gene expression analysis by qRT-PCR. qRT-PCR reactions were carried out as described below with the specific primers for *PPARG2* (Table S1), normalized to endogenous *ACTB* and compared to basal expression levels in non-differentiated cells.

Differentiation control PDMCs were cultured in complete culture medium for 21 days and estimated for adipogenic differentiation by staining with Oil Red O (Sigma, USA) and qRT-PCR as described above.

Osteogenic differentiation

For osteogenic differentiation, cells were treated with osteogenic differentiation medium containing DMEM high glucose (HyClone), 10% FBS (Gibco), 2 mM L-glutamine (Biomedicals), 5 mM HEPES (Biomedicals), 0.1 mM ascorbic acid 2-phosphate (Sigma, USA), 0.1 µM dexamethasone (Sigma, USA), 10 mM β-glycerophosphate (Sigma, USA), 100 U/ml penicillin and 50 µg/ml streptomycin (Sigma, USA). The cells were placed on 6-well plates. When the cells were of 80–90% confluence in a monolayer, the culture medium was changed to osteogenic differentiation medium, and the cells were incubated for 21 days. The differentiation medium was changed twice a week. Alizarin Red S staining was performed at day 21 to assess extracellular calcium deposition. For Alizarin Red S staining, the cells were washed with PBS and fixed with ice-cold 70% ethanol for 5 min at RT. Next, the cells were rinsed three to four times with distilled water and stained with 0.5% Alizarin Red S (pH 4.2, Sigma, USA) for 10 min at RT. The cells were rinsed with distilled water to remove excess dye before observing under the microscope for imaging. PDMCs were pelleted, and RNA was collected after 21 days for osteogenic gene expression analysis byqRT-PCR. qRT-PCR reactions were carried out as described below using specific primers for osteopontin (*SPP1*) (Table S1), normalized to endogenous *ACTB* and compared to basal expression levels in non-differentiated cells. As differentiation control, PDMCs were cultured in complete culture medium for 21 days and estimated for osteogenic differentiation by staining with Alizarin Red S (Sigma, USA) and qRT-PCR as described above.

RNA extraction and RT-PCR

Total RNA was isolated using TRI Reagent (Sigma, USA) and stored at -80 °C. Trace amounts of genomic DNA were removed by RNAse-free DNAse I treatment (Thermo Scientific, USA). Then, cDNA was synthesized from 1 µg of total RNA with oligo(dT)$_{18}$ primers and RevertAid H Minus First Strand cDNA Synthesis Kit (Thermo Scientific, USA) according to the manufacturer's instructions. Two percent of the first-strand cDNA was amplified by sequence-specific primers (Table S1) and Maxima Hot Start Taq DNA polymerase (Thermo Scientific, USA) according to the manufacturer's recommendations. Five microlitres of the resulting product were used for the second round of PCR with nested primers. RT-PCR experiments were repeatedly performed with reproducible results. *ACTB* served as a ubiquitously expressed reference gene. PCR products were analyzed with ethidium bromide-stained 1.2% agarose gel electrophoresis and then sequenced to confirm fidelity. Analysis of the gel images was carried out with ChemiDoc™XRS+System (Bio-Rad, USA), and sequencing was performed on an Applied Biosystems 3130 Genetic Analyzer (Life Technologies, USA). The results were analyzed with BLAST (http://blast.ncbi.nlm.nih.gov/Blast.cgi) and DNASTAR software.

Karyotyping

Chromosome analysis of P3 PDMC cultures from 8 donors was performed. Cells were detached with 0.05% trypsin-EDTA (Biochrom, UK) and then incubated in hypotonic 0.9% (w/v) KCl for 20 min at +37 °C. After fixation with Carnoy's Fixative (3:1 of Methanol: Glacial Acetic Acid), the karyotypes of the PDMCs were determined at the 400-band level of resolution. Cytogenetic results were based on

examination of GTG-banded chromosomes from 15-20 metaphase cells. Slides were examined with a Nikon Eclipse Ni-U microscope (Nikon Corporation, Japan). Images were recorded with a Camera ProgRes MF (Jenoptik, Germany) and processed with Software Lucia Cytogenetics Karyo (Laboratory Imaging, Praha, Czech Republic).

Table S1. Primers sequences and product sizes

Gene ID	Primer forward (5'–3')	Primer reverse (5'–3')	°C	Size (bp)
CDX2 [1]	GGAGTTTCACTACAGTCGCTAC	GAGCCAGACACTGAGGCTTG	57	276
CGB for seq. PCR-product [1]	GCTACTGCCCCACCATGACC	GGATTGAGAAGCCTTTATTGT	62	346
CGB seq.pr. (in this study)	CAGGTGGTGTGCAACTACCGC			
CGB (in this study)	CAGGTGGTGTGCAACTACCGC	GGATTGAGAAGCCTTTATTGT		293
COL2A1[2]	AGTGGAGACTACTGGATTGA	AGTGTACGTGAACCTGCTAT	56	414
VASA/DDX[3]	ACAGGATGTTCCTGCATGGT	TGCCCTTTCTGGTATCAACTG	53	138
EOMES[1]	CACCCCAACAGAGCGAAGAG	AGAGATTTGATGGAAGGGGGTGTC	60	374
ERVW-1[4]	TCATATCTAAGCCCCGCAAC	TGATCTTGCAAGGTGACCAG	57	187
GATA3[5]	AACTGTCAGACCACCACAACCACAC	GGATGCCTTCCTTCTTCATAGTCAGG	57	130
GCM1[6]	CTGAAGGGGAGCACAGAGAC	TCTGTGATTCCTCCCAGACC	54	200
IFITM3 (in this study)	CTTCATAGCATTCGCCTACTCC	CTGATCTATCCATAGGCCTGGA	57	186
POU5F1[7]	AAGCGATCAAGCAGCGACTAT	GGAAAGGGACCGAGGAGTACA	58	132
PPARG2[8]	TGTCAGTACTGTCGGTTTC	AATGGTGATTTGTCTGTTG	56	257
SPP1[9]	CTAGGCATCACCTGTGCCATACC	CAGTGACCAGTTCATCAGATTCATC	57	373
ACTB[1]	GGACTTCGAGCAAGAGAT	AGCACTGTGTTGGCGTAC	57	234

Table S2. Antibodies used for cell characterization by flow cytometry.

N	Name	Manufacturer
1	FITC Anti-Human CD90	BD Pharmingen, USA
2	PerCP-Cy™5.5 Mouse anti-Human CD105 (Endoglin)	
3	PE Mouse Anti-Human CD73	
4	APC Mouse Anti-Human CD34	
5	APC-Cy7 Mouse Anti-Human CD45	
6	Pacific Blue™ Mouse Anti-Human CD14	
7	Rat anti-mouse IgG2a+b PerCP	
8	Monoclonal CD133/1 (AC133) antibodies,human conjugated to PE	MiltenyiBiotec GmbH, Germany
9	Mouse Anti-Human HLA-ABC Common Antigen, Monoclonal antibody	Millipore, USA

Table S3. Antibodies used for cell characterization by immunofluorescence.ICC – immunocytochemistry, IHC – immunohistochemistry.

№	Name	Dilution used for		Manuf.
		ICC	IHC	
1.	Monoclonal Mouse Anti-Human Cytokeratin	1:50	1:50	Dako, Denmark
2.	Monoclonal Mouse Anti-Human Cytokeratin 7	Ready-to-Use	not used	
3.	Rabbit Anti-Human Chorionic Gonadotropin	1:500	1:500	
4.	Monoclonal Mouse Anti-Vimentin	1:200	1:200	
5.	Monoclonal Rabbit Anti-Human ERG (Ets-Related Gene)	1:50	1:50	
6.	Monoclonal Mouse Anti-Human Cytokeratin 18	Ready-to-Use	not used	
7.	Monoclonal Mouse Anti-Human Cytokeratin 19	1:50	not used	
8.	Monoclonal Mouse Anti-Human Actin (Smooth Muscle)	Ready-to-Use	Ready-to-Use	
9.	Monoclonal Mouse Anti-Human CD68	not used	Ready-to-Use	
10.	Monoclonal Mouse Cytokeratin 7 Antibody	1:200	1:200	Novus Biologicals, UK
11.	Monoclonal Rabbit Anti-CDX2 antibody	1:250	1:250	Abcam, UK
12.	Rabbit Anti-TBR2 / Eomes antibody	1:100	1:200	
13.	PCNA (Proliferating Cell Nuclear Antigen) Ab-1, Mouse Monoclonal Antibody	not used	1:200	Thermo Scientific, USA
14.	Goat anti-Mouse IgG (H+L) Secondary Antibody, Alexa Fluor® 488 conjugate	1:1000	1:1000	Life Technologies, USA
15.	Donkey anti-Rabbit IgG (H+L) Secondary Antibody, Alexa Fluor® 555 conjugate	1:1000	1:1000	

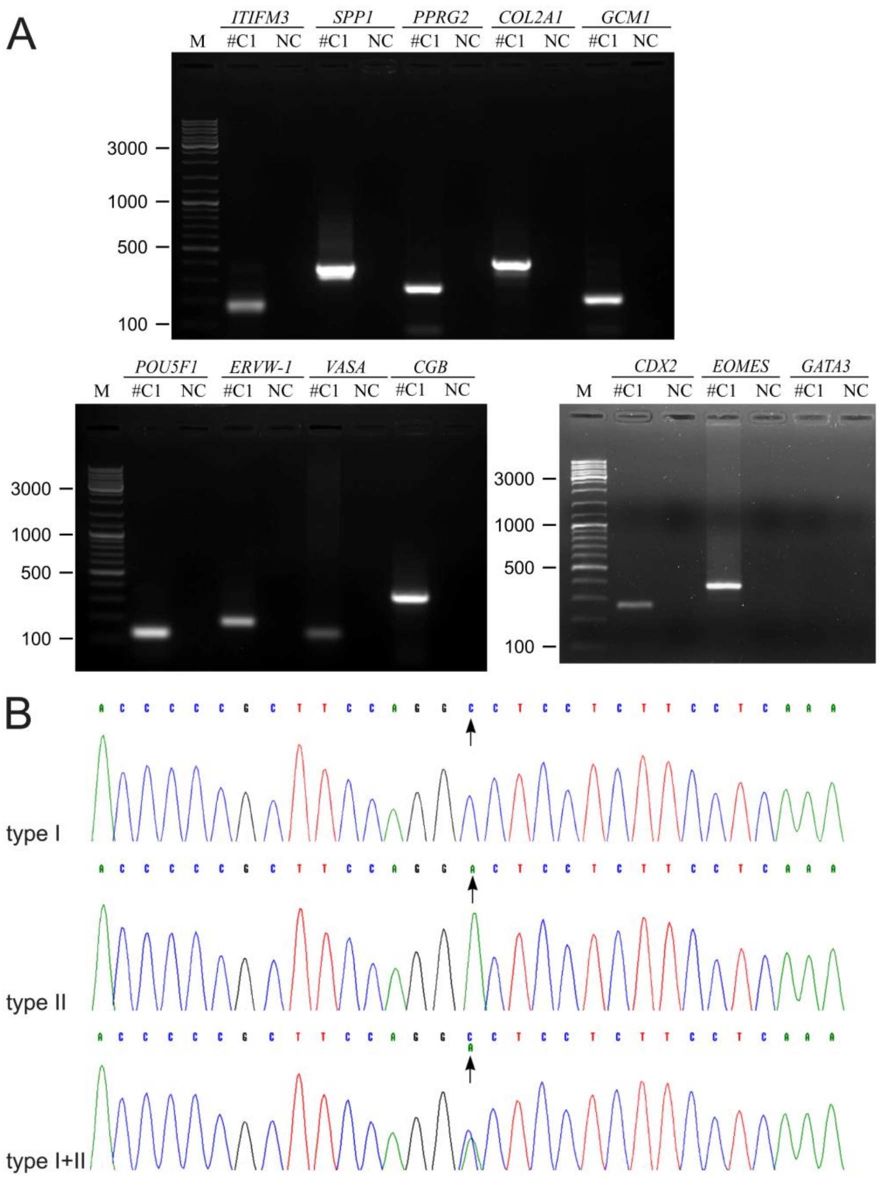

Fig. S1. CK7[low]-clones expressed stemness-, mesoderm- and trophoblast-related genes. (A) A representative example of RT-PCR analysis of PDMC-C1 at P5; M, GeneRuler™ DNA Ladder Mix (Thermo Scientific, USA), NC, water was used as negative control. (B) A part of the nucleotide sequence of A117N (GCC/GAC) in the *CGB* mRNA. Vertical arrow points to the nucleotide differences.

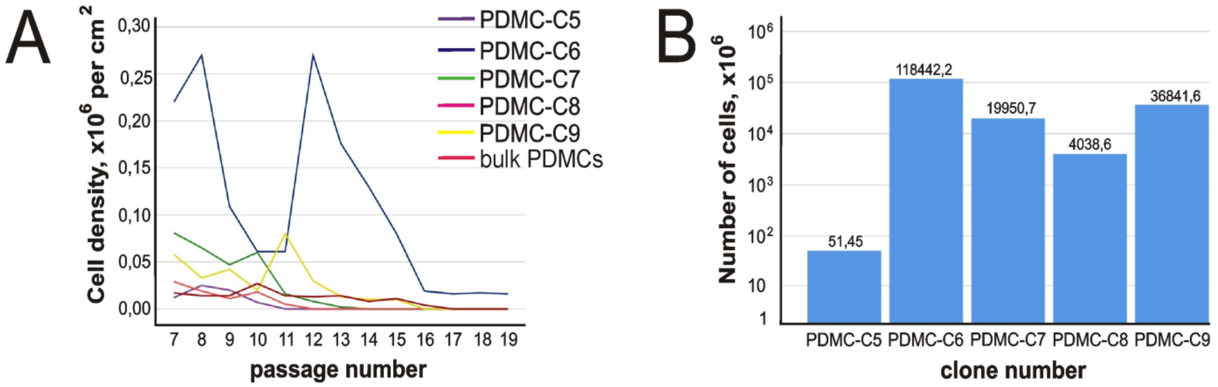

Fig. S2. Proliferative characteristics of CK7^{low}-single cell derived clones. (A) Cell density per cm^2 of culture area after trypsin dissociation at 80% confluence (passages 7-19) for CK7^{low}-clones and bulk PDMCs. (B) Total CK7^{low}-clones output for passage 10. Because only parts of cells were replated at each passage, the cell outputs were calculated assuming all the cells from the previous passage had been replated.

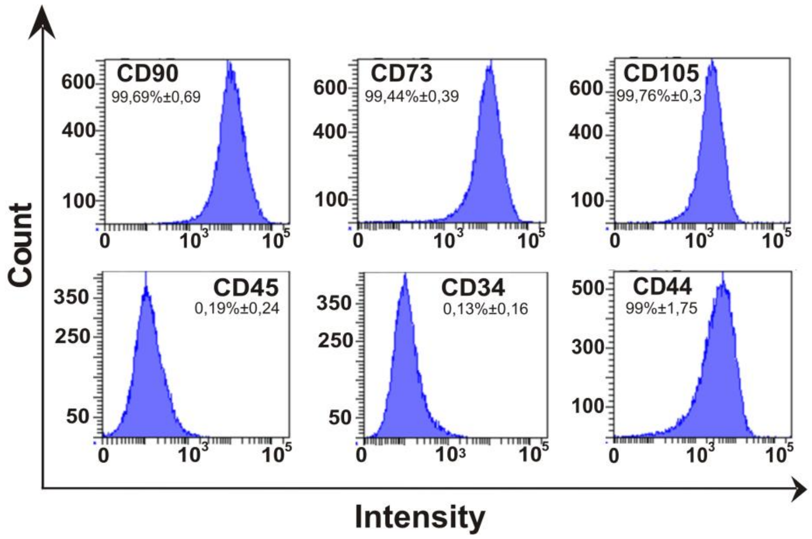

Fig. S3. CK7^{low}- single cell derived clones were positive for CD90, CD73, CD105, CD44 and negative for CD34, CD45 at P3; n=5, M±SD.

Table S4. The gene expression profile of CK7^{low}-clones at P5.

Genes	PDMC-C1	PDMC-C2	PDMC-C3	PDMC-C4	PDMC-C5	PDMC-C6	PDMC-C7	PDMC-C8	PDMC-C9
CDX2	+	+	+	+	+	+	+	+	+
EOMES	+	+	+	+	+	+	+	+	+
POU5F1	+	-	-	-	+	+	+	+	-
VASA	+	-	-	+	-	+	+	-	-
IFITM3	+	+	+	+	+	+	+	+	+
SPP1	+	+	+	+	+	+	+	+	+
COL2A1	+	+	+	+	+	+	+	+	+
PPARG2	+	+	+	+	+	+	+	+	+
GATA3	-	-	-	-	+	+	+	-	-
ERVW1	+	+	+	+	+	+	+	+	+
GCM1	+	-	-	-	-	-	+	-	-
CGB type I	+	-	-	-	+	+	+	+	+
CGB type II	-	+	+	+	+	-	+	+	-

References

[1] H. Svitina, V. Kyryk, I. Skrypkina, M. Kuchma, T. Bukreieva, P. Areshkov, Y. Shablii, Y. Denis, P. Klymenko, L. Garmanchuk, L. Ostapchenko, G. Lobintseva, and V. Shablii, "Placenta-derived multipotent cells have no effect on the size and number of DMH-induced colon tumors in rats," *Exp. Ther. Med.*, vol. 14, no. 3, 2017.

[2] F. Wang, M. Yu, X. Yan, Y. Wen, Q. Zeng, W. Yue, P. Yang, and X. Pei, "Gingiva-Derived Mesenchymal Stem Cell-Mediated Therapeutic Approach for Bone Tissue Regeneration," *Stem Cells Dev.*, vol. 20, no. 12, pp. 2093–2102, Dec. 2011.

[3] V. Schumacher, B. Gueler, L. H. J. Looijenga, J. U. Becker, K. Amann, R. Engers, J. Dotsch, H. Stoop, W. Schulz, and B. Royer-Pokora, "Characteristics of testicular dysgenesis syndrome and decreased expression of SRY and SOX9 in Frasier syndrome," *Mol. Reprod. Dev.*, vol. 75, no. 9, pp. 1484–1494, Sep. 2008.

[4] E. Balestrieri, C. Arpino, C. Matteucci, R. Sorrentino, F. Pica, R. Alessandrelli, A. Coniglio, P. Curatolo, G. Rezza, F. Macciardi, E. Garaci, S. Gaudi, and P. Sinibaldi-Vallebona, "HERVs Expression in Autism Spectrum Disorders," *PLoS One*, vol. 7, no. 11, p. e48831, Nov. 2012.

[5] C. Börner, V. Höllt, and J. Kraus, "Cannabinoid receptor type 2 agonists induce transcription of the mu-opioid receptor gene in Jurkat T cells.," *Mol. Pharmacol.*, vol. 69, no. 4, pp. 1486–91, Apr. 2006.

[6] V. Galat, S. Malchenko, Y. Galat, A. Ishkin, Y. Nikolsky, S. T. Kosak, B. M. Soares, P. Iannaccone, J. D. Crispino, and M. J. C. Hendrix, "A model of early human embryonic stem cell differentiation reveals inter- and intracellular changes on transition to squamous epithelium.," *Stem Cells Dev.*, vol. 21, no. 8, pp. 1250–63, May 2012.

[7] D. S. Moura, I. F. Fernández, G. Marín-Royo, I. López-Sánchez, E. Martín-Doncel, F. M. Vega, and P. A. Lazo, "Oncogenic Sox2 regulates and cooperates with VRK1 in cell cycle progression and differentiation.," *Sci. Rep.*, vol. 6, no. 1, p. 28532, Sep. 2016.

[8] C.-P. Chen, S.-H. Liu, J.-P. Huang, J. D. Aplin, Y.-H. Wu, P.-C. Chen, C.-S. Hu, C.-C. Ko, M.-Y. Lee, and C.-Y. Chen, "Engraftment potential of human placenta-derived mesenchymal stem cells after in utero transplantation in rats.," *Hum. Reprod.*, vol. 24, no. 1, pp. 154–65, Jan. 2009.

[9] R. Kurose, S. Ichinohe, G. Tajima, S. Horiuchi, A. Kurose, T. Sawai, and T. Shimamura, "Characterization of human synovial fluid cells of 26 patients with

osteoarthritis knee for cartilage repair therapy.," *Int. J. Rheum. Dis.*, vol. 13, no. 1, pp. 68–74, Feb. 2010.
